# Efficacy of interventions that use apps to improve diet, physical activity and sedentary behaviour: a systematic review

**DOI:** 10.1186/s12966-016-0454-y

**Published:** 2016-12-07

**Authors:** Stephanie Schoeppe, Stephanie Alley, Wendy Van Lippevelde, Nicola A. Bray, Susan L. Williams, Mitch J. Duncan, Corneel Vandelanotte

**Affiliations:** 1 Central Queensland University, School of Health, Medical and Applied Sciences, Physical Activity Research Group, Building 77, Bruce Highway, Rockhampton, QLD 4702 Australia; 2 Ghent University, Department of Public Health, De Pintelaan 185-4 K3 room 036, 9000 Ghent, Belgium; 3 Central Queensland University, School of Health, Medical and Applied Sciences, Building 6, Bruce Highway, Rockhampton, QLD 4702 Australia; 4 The University of Newcastle, School of Medicine & Public Health, Priority Research Centre for Physical Activity and Nutrition, University Drive, Callaghan, NSW 2308 Australia

**Keywords:** Systematic review, Literature searches, Smartphone applications, Programs, Efficacy, Healthy eating, Exercise, Sitting, Health behaviour

## Abstract

**Background:**

Health and fitness applications (apps) have gained popularity in interventions to improve diet, physical activity and sedentary behaviours but their efficacy is unclear. This systematic review examined the efficacy of interventions that use apps to improve diet, physical activity and sedentary behaviour in children and adults.

**Methods:**

Systematic literature searches were conducted in five databases to identify papers published between 2006 and 2016. Studies were included if they used a smartphone app in an intervention to improve diet, physical activity and/or sedentary behaviour for prevention. Interventions could be stand-alone interventions using an app only, or multi-component interventions including an app as one of several intervention components. Outcomes measured were changes in the health behaviours and related health outcomes (i.e., fitness, body weight, blood pressure, glucose, cholesterol, quality of life). Study inclusion and methodological quality were independently assessed by two reviewers.

**Results:**

Twenty-seven studies were included, most were randomised controlled trials (*n* = 19; 70%). Twenty-three studies targeted adults (17 showed significant health improvements) and four studies targeted children (two demonstrated significant health improvements). Twenty-one studies targeted physical activity (14 showed significant health improvements), 13 studies targeted diet (seven showed significant health improvements) and five studies targeted sedentary behaviour (two showed significant health improvements). More studies (*n* = 12; 63%) of those reporting significant effects detected between-group improvements in the health behaviour or related health outcomes, whilst fewer studies (*n* = 8; 42%) reported significant within-group improvements. A larger proportion of multi-component interventions (8 out of 13; 62%) showed significant between-group improvements compared to stand-alone app interventions (5 out of 14; 36%). Eleven studies reported app usage statistics, and three of them demonstrated that higher app usage was associated with improved health outcomes.

**Conclusions:**

This review provided modest evidence that app-based interventions to improve diet, physical activity and sedentary behaviours can be effective. Multi-component interventions appear to be more effective than stand-alone app interventions, however, this remains to be confirmed in controlled trials. Future research is needed on the optimal number and combination of app features, behaviour change techniques, and level of participant contact needed to maximise user engagement and intervention efficacy.

**Electronic supplementary material:**

The online version of this article (doi:10.1186/s12966-016-0454-y) contains supplementary material, which is available to authorized users.

## Background

The prevention of non-communicable diseases (NCDs) such as cardiovascular disease, cancer and type 2 diabetes is a major public health goal worldwide [1]. In 2012, NCDs were responsible for 38 million (68%) of the world’s 56 million deaths [1]. Lifestyle behaviours including diet, physical activity and sedentary behaviour are key modifiable risk factors for these diseases and improving these behaviours is considered essential to reducing the financial and health burden of these NCDs [1]. Unhealthy diet, physical inactivity and sedentary behaviour are known to track from childhood into adulthood and are difficult to change later in life [2, 3]. This exacerbates associated health problems and demonstrates why preventing the development of these health risk factors throughout the lifespan is important.

Given the global scale of NCDs, preventative interventions that can reach large populations at low cost are needed. Smartphones and tablets, including the software applications (apps) that run on these devices, have become an integral part of people’s life with large increases in usage rates since their introduction in 2007 [4, 5]. For example, 70% of Americans, 61% of Europeans and 74% of Australians regularly use a smartphone and/or tablet [5, 6]. The growth in mobile technologies has also stimulated the growth in health and fitness apps to provide behavioural interventions that can reach large populations [7]. Clever use of apps in health promotion and prevention of disease has enabled researchers to apply health behaviour changes techniques (e.g., goal setting, self-monitoring, performance feedback) that have proven to facilitate health behaviour change across diverse population groups and settings [7, 8].

Despite the increasing use of apps in health behaviour change studies their efficacy is unclear, particularly for different lifestyle behaviours such as diet, physical activity and sedentary behaviour, as well as for specific population groups including children and adults. Some systematic reviews [9–13] have addressed the potential of health apps to improve diet, physical activity and/or sedentary behaviour. However, the scope of these reviews has been broad and many questions remain. Firstly, most previous reviews [9–11, 14] were on electronic and mobile health (e-& mHealth) interventions, not specifically on app-based interventions. Secondly, many reviews [7, 8, 15–18] have focused on app design, the incorporation of established behaviour change techniques and they examined intervention feasibility rather than efficacy. Thirdly, many previous reviews [19–22] have focused on the use of apps for the treatment of obesity and chronic diseases. As such, little is known about the potential of using apps to change health behaviours for disease prevention. Finally, no reviews have distinguished between app interventions for children versus adults. This is important as app features appealing to children and adolescents may differ from those used by adults, and the choice of app features will likely determine user engagement, retention and ultimately intervention efficacy [22].

This review aimed to address these gaps in the literature by systematically synthesising evidence for the efficacy of interventions that use apps to improve diet, physical activity and sedentary behaviour for NCD prevention. Since app features used by children may differ from those used by adults, we distinguished between app interventions targeted to children and adults.

## Methods

### Literature search

This review was conducted and reported according to the Preferred Reporting Items for Systematic Reviews and Meta-Analyses (PRISMA) guidelines (Additional file 1) [23]. Systematic searches were conducted between November and December 2015 in five databases: Scopus, CINAHL, SportDiscuss, PsycINFO and Web of Science. The search was limited to English language literature, humans, and the year of publication between 01 January 2006 and 31 October 2016. It was considered unlikely that app interventions were developed before 2006/2007 when smartphones were introduced. Systematic search strategies were designed using a combination of thesaurus and free terms covering the following terms: application, app, smartphone, smart phone, tablet, mobile game, game, physical activity, walk, physical fitness, leisure activity, motor activity, exercise, sport, sedentary, sedentary behaviour, sedentary behavior, sitting, screen time, inactive, diet, dietary, nutrition, nutritional, healthy eating, food, fruit, vegetable, snack, soft drink, carbonated beverage, intervention, program, programme, health promotion, prevention and trial. The detailed search strategies used for each database are presented in the Additional file 2. Additionally, articles were identified via hand-searching and reviewing reference lists of relevant papers.

### Inclusion criteria and study selection

Studies were included in the present review if (1) they used an app in an intervention to influence at least one of the following lifestyle behaviours: dietary intake, physical activity, sedentary behaviour; (2) targeted children and/or adults; (3) focused on behaviour change for disease prevention (i.e., not specifically aiming to treat and manage health conditions such as obesity, hypertension and NCDs); and (4) reported data regarding efficacy for behaviour change (e.g., change in daily minutes of physical activity). In addition, studies could also report (but were not required) other relevant outcomes that may have conceivably been impacted by health behaviour change (i.e., fitness, body weight, blood pressure, glucose, cholesterol, quality of life). All types and units of measurements for the lifestyle behaviours and related health outcomes were acceptable (e.g., objective measure, self-report, minutes, steps, servings, calories, kilograms). The app intervention could be a stand-alone intervention using apps only, or a multi-component intervention where the use of an app was one of several intervention components (e.g., physical education, provision of physical activity equipment, parental education, face-to-face counselling). Studies were excluded from the review if: (1) non-experimental study designs were utilised (i.e., observational or case studies, studies reporting prevalence or trend data, feasibility studies, measurement studies, theoretical papers); and (2) the publication was not a peer-reviewed primary study (i.e., letters, commentaries, conference proceedings, reviews, narrative articles). Initially, titles and abstracts were screened for inclusion by a single reviewer (SS). As per best practice for systematic reviews [23], two reviewers (SA, WVL) independently reviewed the eligibility of studies for inclusion in the review, with disagreement resolved by discussion and consensus with a third reviewer (SS).

### Data extraction

Data extraction was conducted using a standardised form developed specifically for this review (Table 1); similar to those used in other systematic reviews [12, 24, 25]. For all included studies, data were extracted for author, year, country, study design, intervention duration, measurement time points, attrition rate, sample, behaviour change theory, app features including behaviour change techniques, intervention components, outcomes, measures and main study results. The primary outcome measures extracted for main study results were dietary intake, physical activity and sedentary behaviour. Other relevant outcome measures closely related to these behaviours included weight status (body mass index, body weight, waist circumference), fitness, blood pressure, cholesterol, glucose and quality of life. To determine whether the interventions had a significant effect on behavioural and health outcomes, data on significance and magnitude of within- and between-group differences was extracted. For each included study, two reviewers independently extracted data (NAB and either SS, SA or WVL). Reviewers one and two agreed on the data extraction in over 70% of the studies. Disagreement was easily resolved by discussion and consensus with a third reviewer (either SS, SA or WVL).

**Table 1:** Characteristics of the app-based intervention studies included in the review

Author Year Country	Study design Duration Sample	Behaviour change theory App features Intervention	Outcomes Measures	Results Study quality
Partridge et al. 2015 [31]; Allman-Farinelli et al. 2016 [32] Australia	*Study design* 2-group RCT *Duration* Intervention exposure: 12 weeks (Nov 2012 – July 2014) Measurement points: baseline, 12 weeks, 9 months Attrition rate: 14% *Sample* Adults *N* = 250 (248 analysed) 27.7 years/18-35 years 39% (M), 61% (F) Random	*Behaviour change theory* Transtheoretical model *App features* Newly designed apps: *4 apps (one per behaviour);* self-monitoring of behaviours, educational information, social networking through community blog, informational support resources *Intervention group* Used the apps; received eight text messages and 1 email weekly, five personalised coaching calls, a diet booklet and access to resources and four apps via a website *Control group* Received introductory call at week 0, four text messages in total, and printed dietary and physical activity guidelines *Multi-component versus stand-alone app intervention* Multi-component	*Outcome* Physical activity (MET minutes/week, days/week) Diet (daily fruit and vegetable intake, weekly fast food and sugar-sweetened beverages intake) *Other relevant outcomes* Weight status (body weight, BMI) *Measures* Physical activity (self-report: International Physical Activity Questionnaire) Diet (self-report, questionnaire) Weight status (objectively measured height and weight)	*Diet* At 12 weeks, significant between-group effects in vegetable (*p* = 0.009), fast food (*p* = 0.01) and sugar-sweetened beverages (*p* = 0.002) intake in favour of the intervention group. Significant between-group improvements were sustained at 9 months follow-up. No significant between- group difference in fruit intake. *Physical activity* At 12 weeks, significant between-group increase in total physical activity by 1.3 days/week (95% CI: 0.5–2.2, *P* = 0.003) in intervention group compared to control group. No significant between- group difference at 9 months follow-up. *Weight status* At 12 weeks, significant between-group effects in body weight and BMI: Participants in the intervention group were 2.2 kg lighter compared to the control group (95% CI: 0.8-3.6, *p* =0.005); and they had a 0.5 kg/m2 lower BMI (95% CI: 0.1-1.0, *p* =0.02). Significant between-group improvements were sustained at 9 months follow-up. *Study quality* High CONSORT score: 21 Percentage of fulfilled criteria: 85.7%
Choi et al. 2016 [44] USA	*Study design* 2-group RCT *Duration* Intervention exposure: 12 weeks Measurement points: baseline, 12 weeks Attrition rate: 17% *Sample* Adults *N* = 35 33.7 years/18–40 years 100% (F) Random	*Behaviour change theory* Social cognitive theory *App features* Commercially available app: Fitbit app and tracker; includes self-monitoring of steps, performance feedback, goal-setting *Intervention group* Initial informational/educational session including specific goal setting and receiving IOM recommendations for gestational weight gain and safety instructions for promoting physical activity during pregnancy; used Fitbit app and tracker; tips for physical activity and healthy diet via the app; daily messages (either text or short videos) and activity diary, images and short video clips regarding posture and stretching, all via the app *Control group* Used only the Fitbit tracker, initial brief in-person session, received IOM recommendations for gestational weight gain and safety instruction for promoting physical activity during pregnancy *Multi-component versus stand-alone app intervention* Multi-component	*Outcome* Physical activity (weekly steps counts) *Other relevant outcomes* None *Measures* Fitbit accelerometer Questionnaires	*Physical activity* No significant changes in physical activity. *Study quality* High CONSORT score: 17.5 Percentage of fulfilled criteria: 72.9%
Cowdery et al. 2015 [35] USA	*Study design* 2-group RCT *Duration* Intervention exposure: 12 weeks (Sept- Nov 2014) Measurement points: baseline, 12 weeks Attrition rate: 4% *Sample* Adults *N* = 40 Median 32.0 years/18–69 years 15% (M), 85% (F) Random	*Behaviour change theory* Self-determination theory *App features* Commercially available apps: Gamification via immersive exergame apps (Zombies Run!, The Walk) that instruct users to run and exercise as part of an audio adventure game, self-monitoring and performance feedback via physical activity tracking app (MOVES) that monitors physical activity frequency, duration, intensity and distance *Intervention group* Use of one of the exergame apps, self-monitoring of physical activity through the MOVES app, weekly motivational emails to increase intrinsic motivation for physical activity based on self-determination theory *Control group* Used only physical activity tracking app (MOVES) *Multi-component versus stand-alone app intervention* Multi-component	*Outcome* Physical activity (moderate, vigorous and total physical activity, and walking; minutes/week) *Other relevant outcomes* Weight status (BMI) Blood pressure (diastolic and systolic) *Measures* Accelerometer (physical activity tracking app MOVES) Questionnaires Blood pressure (monitor Omron-BP-760)	*Physical activity* No significant changes in physical activity. *Blood pressure* No significant changes in blood pressure. *Weight status* No significant changes in BMI. *Study quality* Fair CONSORT score: 13.5 Percentage of fulfilled criteria: 56.3%
Direito et al. 2015 [47] New Zealand	*Study design* 3-group RCT *Duration* Intervention exposure: 8 weeks (June-Sept 2014) Measurement points: baseline, 8 weeks Attrition rate: 8% *Sample* Children *N* = 51 15.7 years/14–17 years 43% (M), 57% (F) Random	*Behaviour change theory* Not reported *App features* Commercially available apps: App 1: Immersive exergame app (Zombies, Run!) that provides a training program to improve fitness and ability to run 5 km, information on running and technique, audio instructions on how to perform training components, tracked and displayed progress, social networking App 2: non-immersive app (Get Running) that provides an automated training program to improve fitness and ability to run 5 km, self-monitoring (steps via pedometer), performance feedback via app *Intervention group 1:* Used exergame app (Zombies, Run!) to improve fitness and ability to run 5 km: game-themed whereby the training program was embedded with a story where the user is trained to collect supplies and protect a town from zombies, self-monitoring, performance feedback via app *Intervention group 2:* Used non-immersive app (Get Running) that provides an automated training program to improve fitness and ability to run 5 km, self-monitoring, performance feedback via app *Control group* Doing usual physical activities *Multi-component versus stand-alone app intervention* Stand-alone	*Outcome* Physical activity (daily light, moderate-to-vigorous, total) Sedentary behaviour (total sedentary time minutes/day) *Other relevant outcomes* Cardiorespiratory fitness *Measures* Cardiorespiratory fitness (1 mile run/walk test) Physical activity (questionnaires, accelerometer worn on 7 consecutive days during waking hours)	*Physical activity* No significant changes in light PA, MVPA and overall PA. *Sedentary behaviour* No significant changes in sedentary time. *Fitness* No significant changes in cardiorespiratory fitness. *Study quality* High CONSORT score: 21 Percentage of fulfilled criteria: 87.5%
Elbert et al. 2016 [57] Netherlands	*Study design* 3-group RCT *Duration* Intervention exposure: 6 months Measurement points: baseline, 6 months Attrition rate: 57% Sample Adults *N* = 342 (146 analysed) 41.4 years/16–71 27% (M), 73% (F) Random	*Behaviour change theory* Not reported *App features* Newly designed app: Fruit and Vegetables hAPP: Includes tailored educational information via either text or audio messages, action planning, fruit and vegetable examples and recipes *Intervention group 1* Monthly text-based tailored health information/messages delivered via the app; used all other app features; received unique testimonials (constructed stories) via the app in which successful personal experiences were shared to encourage participants to perform the behaviour themselves *Intervention group 2* Monthly audio-based tailored health information/messages from a female actor delivered via the app; used all other app features; received unique testimonials (constructed stories) via the app in which successful personal experiences are shared to encourage participants to perform the behaviour themselves *Control group* No intervention *Multi-component versus stand-alone app intervention* Stand-alone	*Outcome* Diet (fruit and vegetable intake/servings per week during previous month) *Other relevant outcomes* None *Measures* Diet (Food-Frequency Questionnaire)	*Diet* Main effect analyses: Significant between-group improvement in fruit intake (F2,140 = 3.08, *p* = 0.049: text-based app group (mean servings 13.5, SE 1.0), audio-based app group (mean servings 17.1, SE 1.2), and control group (mean servings 14.3, SE 0.9). However, the significant difference occurred between IG 1(text-based app) and IG2 (audio-based app) (*p* = 0.02), but no significant differences between control group and either of the intervention groups. No significant changes in vegetable intake. Moderation effect analyses: In adults with poor perceived own health status, significant-between-group differences in fruit intake in favour of IG2 (F2,137 = 6.05, *p* = 0.003, partial η2 = 0.08); mean fruit servings were (IG1: 14.2), (IG2: 20.5) and (CG: 13.2). Fruit intake was significantly higher in IG2 compared to IG1 (*p* = 0.006) and CG (*p* = 0.001). Study quality Fair CONSORT score: 14.5 Percentage of fulfilled criteria: 60.4%
Fukuoka et al. 2010 [36] USA	*Study design* Pre-post within-subjects design *Duration* Intervention exposure: 3 weeks (June-Sept 2008) Measurement points: Baseline, 3 weeks Attrition rate: 0% *Sample* Adults *N* = 41 48.0 years/25–40 years 100% (F) Convenience	*Behaviour change theory* Not reported *App features* Newly designed app: Goal setting, self-monitoring of physical activity (step diary), motivational messages *Intervention group* Used the app, used pedometer, received daily prompts regarding benefits of physical activity and social support options *Control group* None *Multi-component versus stand-alone app intervention* Multi-component	*Outcome* Physical activity (daily steps, daily aerobic steps and kcal/kg/day) *Other relevant outcomes* None *Measures* Physical activity (pedometer and self-reported questionnaire: 7-day Physical Activity Recall)	*Physical activity* Significant increase in mean daily steps from baseline (5394; 95% CI: 4563–6224) to 3 weeks (6210; 95% CI: 5379–7041) (*p* = 0.001). Significant increase in mean aerobic steps from baseline (953; 95% CI: 489–1416) to 3 weeks (1535; 95% CI: 1074–1996) (*p* < 0.001). Significant increase in kcal/kg/day from baseline (32.5 ± 1.28) to 3 weeks (33.4 ± 1.99) (*p* = 0.01). *Study quality* Low CONSORT score: 11.5 Percentage of fulfilled criteria: 46.9%
Garde et al. 2015 [37] Canada	*Study design* 2-group CT *Duration* Intervention exposure: 1 week Measurement points: baseline, during intervention Attrition rate: 13% *Sample* Children *N* = 54 (*n* = 47 analysed) 10.0 years/8–13 years 16% (M), 84% (F) Convenience	*Behaviour change theory* Self-determination theory *App features* Newly designed app: App (Mobilekids Monster Manor), physical activity earns gaming currency (gamification), inter-team competition, peer support *Intervention group* Used the app *Control group* Received daily physical activity feedback via a website *Multi-component versus stand-alone app intervention* Stand-alone	*Outcome* PA (steps/day, minutes/day) *Other relevant outcomes* None *Measures* Physical activity (accelerometer-based activity monitor Tractivity)	*Physical activity* No significant between-group changes in physical activity. Significant within-group increase in IG in relation to steps/day (1191; *p* = 0.01) and active minutes/day (25; *p* = 0.03). *Study quality* Fair CONSORT score: 16 Percentage of fulfilled criteria: 65.3%
Gasser et al. 2015 Switzerland	*Study design* 2-group RCT *Duration* Intervention exposure: 4 weeks Measurement points: baseline, 4 weeks Attrition rate: not reported *Sample* Children and adults *N* = 40 (39 analysed) 32.0 years/14–50 years 50% (M), 50% (F) Random (but age and gender controlled)	*Behaviour change theory* Not reported *App features* Newly designed app: Self-monitoring of physical activity and food consumption (via diary in app), goal-setting, individual and team performance feedback on daily goal achievement, social support (teams), received messages, reminders and questionnaires *Intervention group* Used smartphone app *Control group* Used a similar web-based app that worked on any operating system/browsers *Multi-component versus stand-alone app intervention* Stand-alone	*Outcome* Physical activity (daily moderate-to-vigorous) Diet (fruit and vegetable consumption, servings/day) *Other relevant outcomes* Weight status (BMI) *Measures* Online questionnaire	*Diet* No significant changes in fruit and vegetable consumption. *Physical activity* No significant changes in physical activity. *Weight status* No significant changes in BMI. *Study quality* Low CONSORT score: 8.5 Percentage of fulfilled criteria: 34.0%
Gilliland et al. 2015 [38] Canada	*Study design* Pre-post within-subjects design *Duration* Intervention exposure: baseline, 8–10 weeks Measurement points: Baseline, post intervention (varied weeks post baseline) Attrition rate: 44% *Sample* Adults *N* = 208 33.0 years/age range not reported 34% (M), 66% (F) Convenience	*Behaviour change theory* Not reported *App features* Newly designed app: App (SmartAPPetite) that includes education on diet and health, goal setting, rewards, motivational interviewing, time management tips, healthy eating tips, recipes, vendor spotlights and coupons, behaviour-health link *Intervention components* Used the app *Control group* None *Multi-component versus stand-alone app intervention* Stand-alone	*Outcome* Diet (weekly consumption of health food items) *Other relevant outcomes* None *Measures* Diet (self-report questionnaire)	*Diet* Significant correlation between app use (check-ins) and food consumption (vegetables *r* = 0.23; soft drinks *r* = −0.30, fruit juice *r* = −0.35; *p <* 0.05). *Study qualit*y Low CONSORT score: 8.5 Percentage of fulfilled criteria: 34.7%
Gilson et al. 2016 [51] Australia	*Study design* Pre-post within-subjects design *Duration* Intervention exposure: 20 weeks Measurement points: Baseline, weeks 4, 8, 12, 16, 20 Attrition rate: 57% *Sample* Adults *N* = 44 (26 analysed) 47.0 years/age range not reported 100% (M) Convenience	*Behaviour change theory* Not reported *App features* Commercially available app: Jawbone Up that includes self-monitoring of daily step counts and logging dietary choices; includes news feeds, notifications and status updates, can connect with other users *Intervention components* Used the app and received intervention guidance and support by the researchers through connecting via the app *Control group* None *Multi-component versus stand-alone app intervention* Stand-alone	*Outcome* Diet (healthy dietary choices) Physical activity (daily/weekly step counts) *Other relevant outcomes* None *Measures* Diet (self-report questionnaire)	*Diet* No significant changes in healthy diet choices. *Physical activity* No significant changes in step counts. *Study quality* Low CONSORT score: 9.5 Percentage of fulfilled criteria: 39.6%
Glynn et al. 2014 [53] Ireland	*Study design* 2-group RCT *Duration* Intervention exposure: 8 weeks (Aug 2012 - June 2103) Measurement points: baseline, 8 weeks Attrition rate: 14% Sample Children and adults *N* = 90 (77 analysed) 44.1 years/>16 years 36% (M), 64% (F) Random	*Behaviour change theory* Not reported *App features* Commercially available app: Used the Accupedo-Pro Pedometer app. Goal setting functionality and goal setting achievement feedback, self-monitoring of step counts and calories burnt, automatic performance feedback through graphic display of step-count history *Intervention group* Received physical activity goals (10,000 steps/day) and information on the benefits of exercise, smartphone app and instruction on how to use it, telephone mentoring sessions with physical activity goal setting *Control group* Received physical activity goals (walking for 30 min/day in addition to normal activity) and information on benefits of exercise but app not made visible on their smartphone and no instructions on how to use the app to achieve these goals *Multi-component versus stand-alone app intervention* Multi-component	*Outcome* Physical activity (steps/day) *Other relevant outcomes* Weight status (body weight, BMI) Quality of life Blood pressure (diastolic and systolic) *Measures* Physical activity (pedometer) Weight status (objectively measured height and weight) Blood pressure (monitor) Quality of life (questionnaires)	*Physical activity* Significant between-group increase in mean steps/day in IG at 8 week follow-up (1631 ± 3842; *p* = 0.03). *Weight status* No significant changes in body weight. No significant changes in BMI. *Blood pressure* No significant changes in blood pressure. *Quality of life* No significant changes in quality of life. *Study quality* High CONSORT score: 18 Percentage of fulfilled criteria: 73.5%
Hebden et al. 2014 [48] Australia	*Study design* 2-group RCT *Duration* Intervention exposure: 12 weeks (July- Dec 2011) Measurement points: baseline, weeks 13 Attrition rate: 10% *Sample* Adults *N* = 51 23.0 years/18–35 years 20% (M), 80% (F) Random	*Behaviour change theory* Transtheoretical model *App features* Newly designed apps: *4 apps (one per behaviour);* physical activity self-monitoring, servings of fruit and vegetables, energy and fat content of take away meals and tailored advice *Intervention group* Used the apps, received SMS text and email messages and internet forums *Control group* Printed diet booklet with instructions from dietician. *Multi-component versus stand-alone app intervention* Multi-component	*Outcome* Physical activity (light, MET; minutes/week) Sedentary behaviour (sedentary time; minutes/week) Diet (daily fruit and vegetable intake and weekly fast food consumption) *Other relevant outcomes* Weight status (body weight, BMI) *Measures* Physical activity, sedentary behaviour (self-report: International Physical Activity Questionnaire; accelerometer) Sitting time (self-report, questionnaire) Diet (self-report, takeaway and fruit and vegetable consumption) Weight status (objectively measured height and weight)	*Diet* No between- group change in fruit and vegetable intake or consumption of takeaway meals. *Physical activity* Significant between-group increase in light intensity activity in IG at 13 week follow-up (34.2 ± 35.1, *p* = 0.001). No between group differences for self-reported MET minutes of physical activity. *Sedentary behaviour* No significant changes in sedentary behaviour. *Weight status* No significant changes in weight status. *Study quality* High CONSORT score: 19.5 Percentage of fulfilled criteria: 79.6%
King et al. 2013 [39] USA	*Study design* 3-group randomised trial *Duration* Intervention exposure: 8 weeks Measurement points: baseline, 8 weeks Attrition rate: 11% *Sample* Adults *N* = 68 (N 61 analysed) 59.1 years/>45 years 26% (M), 74% (F) Random	*Behaviour change theory* Social cognitive theory, social influence theory *App features* Newly designed apps: 3 different apps: *‘Analytic’* motivational app including goal-setting and feedback, barriers *‘Social’* motivational app including social norms, modelling, competition and collaboration. *‘Affective’* motivational app including positive reinforcement, modelling, feedback and gamification. In addition, all apps incorporated push and pull components, glance-able display, passive activity assessment, real time feedback, self-monitoring, reinforcement. *Intervention group* Used the apps *Control group* None *Multi-component versus stand-alone app intervention* Stand-alone	*Outcome* Physical activity (brisk walking, moderate-to-vigorous; minutes/week) Sedentary behaviour (television viewing; minutes/day) *Other relevant outcomes* None *Measures* Physical activity (self-report: CHAMPS Physical Activity Questionnaire) Sedentary behaviour (self –report: Measure of Older Adults Sedentary Time MOST)	*Physical activity* Significant within-group increases in mean minutes/week of brisk walking across all 3 app groups at 8 week follow-up (100.8 ± 167.0; *p* < 0.001). Significant within-group increase in mean minutes/week of total MVPA across all 3 app groups at 8 week follow-up (188.6 ± 289.3; *p* < 0.001) No significant between-group changes in physical activity. *Sedentary behaviour* Significant within-group decrease in minutes/day spent sitting whilst watching television (29.1 ± 84.5; *p* < 0.02) across all 3 app groups at 8 week follow-up. No significant between-group changes in sedentary behaviour. *Study quality* Low CONSORT score: 12 Percentage of fulfilled criteria: 46.9%
Kirwan et al. 2012 [49] Australia	*Study design* 2-group CT (matched case-control trial) *Duration* Intervention exposure: 12 weeks (August-October 2009) Measurement points: baseline, 12 weeks Attrition rate: 0% Sample Adults *N* = 200 39.7 years/17–64 years 52% (M), 48% (F) Convenience	*Behaviour change theory* Not reported *App features* Newly designed app: Self-monitoring of physical activity (steps via iSteplog) *Intervention group* Participants logged steps using either app or 10,000 steps website, goal-setting, performance feedback *Control group* Participants logged steps using 10,000 step website, but no access to iSteplog app *Multi-component versus stand-alone app intervention* Multi-component	*Outcome* Physical activity (steps/day) *Other relevant outcomes* None *Measures* Accelerometer	*Physical activity* Between group increase in steps/day in IG at 12 week follow-up (11,140. ± 4,121vs CG: 6,274 ± 2,106, *p* < 0.001). *Study quality* Fair CONSORT score: 14.5 Percentage of fulfilled criteria: 59.2%
Maher et al. 2015 [50] Australia	*Study design* 2-group RCT *Duration* Intervention exposure: 8 weeks (September 2013 - July 2014) Measurement points: baseline, 8 weeks, 20 weeks Attrition rate: 13% Sample Adults *N* = 110 35.6 years/18–65 years 29% (M), 71% (F) Random	*Behaviour change theory* Theory of planned behaviour, fun theory *App features* Newly designed app: Facebook app (Active Team) including goal setting (10,000 steps/day), self-monitoring of physical activity (calendar to log daily steps), performance feedback via tally board to monitor individual and teammates’ progress; team message board to allow team members to communicate with one another; gamification in the form of awards for individual and team step-logging and step-count achievement, as well as sending virtual gifts to teammates; peer social support through Facebook friends (Active Teams) *Intervention group* Used the app, automated computer-tailored emails to summarise progress and encourage continued participation, use of pedometer to encourage achieve 10,000 steps/day *Control group* Wait-list control *Multi-component versus stand-alone app intervention* Multi-component	*Outcome* Physical activity (moderate, vigorous, walking; minutes/week) *Other relevant outcomes* Quality of life *Measures* Questionnaires	*Physical activity* 8-week follow-up: Significant between-group increase in mean weekly minutes of overall PA in IG (528 ± 391 vs CG: 391 ± 371, effect size: 0.39, 95% CI:0.01–0.76) and walking (332 ± 289 vs CG: 160 ± 185, effect size: 0.69, 95% CI: 0.30–1.07) 20-week follow-up: Physical activity remained higher compared to baseline, and higher in IG compared to CG. But within-group and between-group differences were not significant. *Quality of life* No significant changes in quality of life at 8-week and 20-week follow-ups. *Study quality* High CONSORT score: 19 Percentage of fulfilled criteria: 77.6%
Mummah et al. 2016 [45] USA	*Study design* 2-group RCT *Duration* Intervention exposure: 12 weeks Measurement points: baseline, 12 weeks Attrition rate: 24% *Sample* Adults *N* = 17 42.05 years/18–50 years 35% (M), 65% (F) Random	*Behaviour change theory* Behavioural theory *App features* Newly designed app: Goal setting for and self-monitoring of vegetable consumption (i.e., vegetable logging by tapping on different vegetable icons and recording the number of servings consumed); performance feedback via graphs, social comparison with friends via leaderboard, consumption challenges delivered via push notifications, prompts to log vegetables via push notifications *Intervention group* Used the app *Control group* Wait-list control *Multi-component versus stand-alone app intervention* Stand-alone	*Outcome* Diet (daily vegetable consumption/servings) *Measures* Questionnaires (Food Frequency Questionnaire)	*Diet* Significant between-group increase in vegetable consumption in intervention group compared to control group (adjusted mean difference: 7.4 servings; 95% CI: 1.4–13.5; *p* = 0.02) *Study quality* High CONSORT score: 17.5 Percentage of fulfilled criteria: 72.9%
Nollen et al. 2014 [40] USA	*Study design* 2-group RCT *Duration* Intervention exposure: 12 weeks (weeks 1–4: fruits/vegetables; weeks 5–8: sugar-sweetened beverages; weeks 9–12: screen time) March 2011- April 2012 Measurement points: baseline, 4 weeks (fruits/vegetables), 8 weeks (sugar-sweetened beverages), 12 weeks (screen time) Attrition rate: 14% *Sample* Children N = 51 11.3 years/9-14 years 100% (F) Random	*Behaviour change theory* Not reported *App features* Commercially available app: Real-time goal setting, action planning, self-monitoring and tips, feedback and positive reinforcements on goal-attainment through song-based rewards system (received 1 song/day if girls responded to 80% of daily prompts) *Intervention group* Used the app *Control group* Used the app but without action cues and reward system *Multi-component versus stand-alone app intervention* Stand-alone	*Outcome* Diet (fruit and vegetable consumption, sugar-sweetened beverages consumption) Sedentary behaviour (screen time) *Other relevant outcomes* Weight status (BMI) *Measures* Diet (questionnaires: 24-h dietary recall) Sedentary behaviour (questionnaires: Brief Questionnaire of Television and Computer use) BMI (objectively measured height and weight)	*Diet* Between-group increase in fruit and vegetable consumption in IG at 12 week follow-up, but not significant (*p* = 0.08). Between-group decrease in sugar-sweetened beverage consumption in IG at 12 week follow-up, but not significant (*p* = 0.09). *Sedentary behaviour* No significant changes in sedentary behaviour. *Weight status* No significant changes in weight status. *Study quality* Fair CONSORT score: 12.5 Percentage of fulfilled criteria: 51.0%
Rabbi et al. 2015 [46] USA	*Study design* 2-group RCT *Duration* Intervention exposure: 3 weeks (randomisation after week 1) Measurement points: baseline, week 3 Attrition rate: 6% *Sample* Adults *N* = 18 (17 analysed) 28.3 years/18-49 years 53% (M), 47% (F) Random	*Behaviour change theory* Learning theory, social cognitive theory, fogg behaviour model *App features* Newly designed app: MyBehaviour app included self-monitoring of physical activity, and food and caloric intake; logging clusters/patterns of physical activities and foods; prompting goal setting via automatic generation of suggestions for exercise and food based on logged activities and food items. *Intervention group* Used the app, received ‘tailored’ suggestions for exercise and food intake via the app based on logged activities and food items; face-to-face training session on how to use the app *Control group* Used the app; received ‘generic’ prescriptive recommendations for physical activities and dietary intake created by health professionals and delivered via the app; face-to-face training session on how to use the app *Multi-component versus stand-alone app intervention* Stand-alone	*Outcome* Diet (caloric intake) Physical activity (walking minutes/week) *Other relevant outcomes* None *Measures* Physical activity and diet (daily diary)	*Diet* No significant changes in diet. *Physical activity* No significant changes in physical activity. *Study quality* Fair CONSORT score: 14 Percentage of fulfilled criteria: 58.3%
Rospo et al. 2016 [56] Italy	*Study design* 3-group randomised controlled trial (only IG1 and IG2 were randomised) *Duration* Intervention exposure: 2 weeks Measurement points: baseline, week 1, week 2 Attrition rate: 27% *Sample* Adults *N* = 45 (33 analysed) 56.6 years/20–55 years 39% (M), 61%(F) Random	*Behaviour change theory* Not reported *App features* Newly designed app vs commercially available app: Both apps included self-monitoring, performance feedback, goal setting. The newly designed cardio fitness app focused on heart rate monitoring in particular *Intervention groups* IG1: Step-count app group Used the Fitbit app, instructed to complete 10,000 steps a day IG2: Cardio fitness app group Used the newly designed cardio fitness app to receive performance feedback, completed an fitness intensity training based on the guidelines of the American College of Sports Medicine IG3: Supervised cardio fitness group Completed an fitness intensity training 3–4 times/week at the gym based on the guidelines of the American College of Sports, received face-to-face performance feedback *Control group* None *Multi-component versus stand-alone app intervention* Stand-alone	*Outcome measures* Physical activity (steps/week) *Other relevant outcomes* Cardiorespiratory fitness (maximal oxygen uptake) Weight status (BMI kg/m^2^) Blood pressure (diastolic and systolic; mm Hg) *Measurements* Physical activity (pedometer) Cardiorespiratory fitness (laboratory tests: Ruffier-Dickson squat test, Ebbeling single-stage treadmill walk test) Weight status (objectively measured weight and height) Blood pressure (measure n.r.)	*Physical activity* Significant between-group improvement in week mean step counts in favour of the non-app Super-CF group (F(2;60) = 4.903, *p* < 0.01), compared to CF-App group at week 2. Super-CF: 9764 steps vs CF-App: 7775 steps; *p* < 0.05. *Cardiorespiratory fitness* Significant within-group improvements in maximal oxygen uptake in all three groups (Step-App: +0.95 mL/kg/min; CF-App: +1.70 mL/kg/min; and Super-CF: +1.85 mL/kg/min). No significant between-group changes. *Weight status* No significant changes in weight status. *Blood pressure* Within-group improvements in systolic (F(1;30) = 4.946, *p* = 0.03; Step-App: +1.19 mm Hg; CF-App: −3.23 mm Hg; Super-CF: −5.75 mm Hg) and diastolic blood pressure (F (1;30) = 12.585, *p* < 0.001; Step-App: −2.12 mm Hg; CF-App: −4.31 mm Hg; Super-CF: −3.54). No significant between-group changes. *Study quality* Fair CONSORT score: 12 Percentage of fulfilled criteria: 50.0%
Safran Naimark et al. 2015 [58] Israel	*Study design* 2-group RCT *Duration* Intervention exposure: 14 weeks (2010–2011) Measurement points: baseline, 14 weeks Attrition rate: 14% *Sample* Adults *N* = 99 47.9 years/≥18 years (age range not reported) 36% (M), 64% (F) Random	*Behaviour change theory* Control systems theory of self-regulation *App features* Newly designed app: eBalance app that includes goal setting, self-monitoring of physical activity, dietary intake, and calorie intake and expenditure; real-time performance feedback; information on nutrient intake compared to dietary recommendations. *Intervention group* Used the app, initial face-to-face information session on healthy lifestyle *Control group* Information session on healthy lifestyle only, instructed to continue living a healthy lifestyle as they understood it *Multi-component versus stand-alone app intervention* Multi-component	*Outcome* Physical activity (minutes/week) Diet (diet quality score) *Other relevant outcomes* Weight status (body weight in kilogram, BMI) BMI *Measures* Physical activity and diet (questionnaire) Weight status (objective height and weight)	*Diet* Significant between-group improvement in diet quality score in IG at 14 week follow-up (+71 ± 7.6; *p* < 0.001). *Physical activity* Significant between-group increase in mean minutes/week of physical activity in IG at 14 week follow-up (+63.0 ± 20.8; *p* = 0.02). *Weight status* Significant between-group decrease in body weight (kg) in IG at 14 week follow-up (−1.44 ± 0.40; *p* = 0.03). Significant between-group decrease in BMI in IG at 14 week follow-up (−0.48 k/m^2^ ± 0.13; *p* = 0.03). *Study quality* High CONSORT score: 16.5 Percentage of fulfilled criteria: 67.3%
Silveira et al. 2013 [33] Van Het Reve et al. 2014 [34] Switzerland	*Study design* 3-group CT *Duration* Intervention exposure: 12 weeks Measurement points: baseline, 12 weeks Attrition rate: 25% *Sample* Older adults *N* = 44 75.0 years/> 65 years 36% (M), 64% (F) Convenience (participants in the intervention groups were randomised but not participants in the control group)	*Behaviour change theory* Motivation theory, Transtheoretical model *App features* Commercially available app: ActiveLifestyle app that includes autonomous strength-balance physical training for independently living older adults. A strength-balance training plan with three levels: beginner, intermediate, and expert. Individual motivation strategies: positive and negative reinforcement, goal setting, self-monitoring, awareness. Social motivation strategies: social comparison, monitoring of peers, emotional support, collaboration with peers to reach common in-game goals. Additional features: a virtual training plan community and communication features (i.e., private text messaging in the app, a bulletin board with links to newspapers, videos, and websites) *Intervention group* IG 1: Individual group that followed training using the individual version of ActiveLifestyle (=individual motivations strategies); IG 2: Social group that followed training using the social version of the ActiveLifestyle app (= the individual and social motivation strategies, the virtual training plan community and communication features) *Control group* Followed exercises with printed information without additional motivation strategy. *Multi-component versus stand-alone app intervention* Stand-alone	*Outcome* Physical activity (gait speed: preferred and fast walking speed) *Other relevant outcomes* None *Measures* Physical activity (gait speed by GAITRite walkway)	*Physical activity* Significant within-group increase in preferred gait speed across all groups at 12-week follow-up (*p* < .001). However, no significant between-group changes. Significant within-group and between-group increases in gait speed at 12-week follow-up. Participants walked significantly faster at post-test (1.72 m/s) than at pre-test (1.56 m/s; *F* =20.1, *p* < .001, ç =0.41). The main effect of group was also significant (*F* =5.3, *p* = .01 ç =0.27). The individual group (1.89 m/s) was significantly faster than the control group (1.45 m/s; t =3.94, *p* = .003, d = 1.31), and faster than the social group (1.58 m/s; t =2.05, *p* = .08, d = .89), though the latter did not reach statistical significance. *Study quality* Low CONSORT score: 12 Percentage of fulfilled criteria: 48.0%
Smith et al. 2014 [29], Lubans et al. 2016 [30] Australia	*Study design* 2-group cluster RCT *Duration* Intervention exposure: 20 weeks (Dec 2012 - June 2013) Measurement points: baseline, 8 months (post-intervention), 18 months Attrition rate: 19% *Sample* Children *N* = 361 12.7 years/12–14 years 100% (M) Random	*Behaviour change theory* Self-determination theory, social cognitive theory *App features* Newly designed app: Goal setting for physical activity and screen time, self-monitoring (uploading pedometer measured steps), tailored motivational and informational messages via ‘push prompts’, assessment of resistance training skill competency, recording fitness challenge results, resistance training and aerobic exercises. *Intervention group* Goal setting, self-monitoring (steps through pedometer), fitness challenge during school sport sessions, teacher professional development, provision of fitness equipment to schools, face-to-face physical activity sessions led by teachers, lunchtime student mentoring sessions, researcher-led educational sessions for children, a smartphone application and website, parental education and tips for reducing screen time through newsletter *Control group* Usual practice (regular school sports and PE lessons) *Multi-component versus stand-alone app intervention* Multi-component	*Outcome* Physical activity (moderate-to-vigorous, total; minutes/day) Sugar-sweetened beverages consumption (glasses/day) *Other relevant outcomes* Fitness Weight status (BMI, waist circumference, body fat) *Measures* Accelerometer (worn on 7 consecutive days including weekend)	*Diet* Significant between-group decrease in mean glasses/day of sugar-sweetened beverage consumption in IG (−0.6 ± 0.26; *p* = 0.01) at 8-months follow-up. No significant intervention effects at 18-months follow-up. *Physical activity* No significant changes in daily MVPA or overall PA at 8-months and 18-months follow-ups. *Sedentary behaviour* Significant between-group difference in mean minutes/day screen-time in favour of IG at 8-months follow-up (−30.0 ± 10.08; *p* = 0.03) and 18-months follow-up (−32.2; 95% CI: −53.6- -10.8; *p* = 0.03). *Fitness* Significant between-group increase in muscular endurance in IG as measured by mean push-ups repetitions (0.9 ± 0.49; *p* =0.04) and resistance training skills (mean units 5.7 ± 0.67; *p* <0.001) at 8–months follow-up. Intervention effect was sustained for resistance training skills at 18-months follow-up (mean units 5.9, 95% CI: 4.5-7.3; *p* < 0.001) *Weight status* No significant intervention effects for BMI, waist circumference and percent of body fat at 8-months and 18-months follow-ups. *Study quality* High CONSORT score: 21.5 Percentage of fulfilled criteria: 91.5%
Stuckey et al. 2011 [41] Canada	*Study design* Pre-post within-subjects design *Duration* Intervention exposure: 8 weeks Measurement points: baseline, week 4, week 8 Attrition rate: 8% *Sample* Adults *N* = 26 56.6 years/30–71 years 25% (M), 75%(F)	*Behaviour change theory* Transtheoretical model *App features* Commercially available app: Self-monitoring: a Smartphone received via Bluetooth info from a blood pressure monitor, a glucometer, and a pedometer. Weight was manually entered. Smartphones transmitted self-monitoring measurements to the database and allowed participants to interface with the researchers as well as view graphical outputs of their personal health indicators. *Intervention group* App intervention plus tailored counselling (every 4 weeks) regarding physical activity and lifestyle modifications with personal goal setting. Participants received a stage-matched activity booklet addressing self-efficacy, decisional balance, and stage-appropriate processes of change (a 2 month data plan). *Control group* None *Multi-component versus stand-alone app intervention* Multi-component	*Outcome measures* Physical activity (steps/day, vo2max) *Other relevant outcomes* Weight status (BMI kg/m^2^, waist circumference) Blood pressure (diastolic and systolic; mm Hg) Blood glucose Cholesterol (LDL, HDL, total, triglycerides; mmol/liter) *Measurements* Physical activity (STEP test) Weight status (objectively measured weight and height) Blood pressure (sphygmomanometer) Blood glucose and cholesterol (venepuncture)	*Physical activity* Significant increase in steps/day in IG at 8-week follow-up (+1,086 ± 1613, *p* = 0.003). Significant increase in vo2max (ml/kg/min) at 8-week follow-up (+5.139 ± 4.911, *p* < 0.001). *Weight status* Significant reduction in BMI in IG at 8-week follow-up (−0.465 ± 0.987, *p* = 0.002). *Blood Glucose* No significant changes. *Blood pressure* Significant reduction in diastolic blood pressure in IG at 8-week follow-up (−4.375 ± 5.640, *p* = 0.001) *Total cholesterol* Significant reduction in total in cholesterol levels in IG at 8-week follow-up (−0.295 ± 0.508, *p* = .009). *Study quality* Low CONSORT score: 11 Percentage of fulfilled criteria: 45.0%
Van Drongelen et al. 2014 [54] The Netherlands	*Study design* 2-group RCT *Duration* Intervention exposure: not reported Measurement points: baseline, 3 months, 6 months Attrition rate: 13.5% *Sample* Adults *N* = 502 40.9 ± 8.4 years/age range: not reported 93% (M), 7% (F) Random	*Behaviour change theory* Not reported *App features* Commercially available app: The MORE Energy app contained evidence-based advice tailored to flight schedules and personal characteristics aiming to reduce fatigue and circadian disruption as much as possible. *Intervention group* The MORE Energy app + a website containing more background information was developed alongside the smartphone app. *Control group* The participants allocated to the control group received a minimal intervention consisting of access to a secure part of the project website, which contained basic, non-tailored, fatigue and health-related information that was already available within the airline company (such as information about sleep hygiene and the working mechanisms of the biological clock). *Multi-component versus stand-alone app intervention* Multi-component	*Outcome* Physical activity (moderate, vigorous; days/week) Diet (breakfast, meal composition, snacking, hydration, caffeine intake) Other relevant outcomes None *Measures* Self-report, questionnaires	*Diet* Significant between-group improvement in snacking behaviour in IG at 6-months follow-up (β = −0.81, *p* < 0.001). *Physical Activity* Significant between-group increase in vigorous physical activity in IG at 6-months follow-up (β = 0.17, *p* = 0.028) *Study quality* High CONSORT score: 17 Percentage of fulfilled criteria: 68.0%
Walsh et al. 2016 [55] Ireland	*Study design* 2-group RCT *Duration* Intervention exposure: 5 weeks Measurement points: baseline, 5 weeks Attrition rate: 5% *Sample* Adults *N* = 58 (55 analysed) 20.55 years/17–26 years 27% (M), 73% (F) Random	*Behaviour change theory* Capability, Opportunity, Motivation, Behaviour (COM-B) framework, Behavior Change Wheel *App features* Commercially available app: The ‘Accupedo-Pro’ pedometer app includes goal setting, self-monitoring, performance feedback *Intervention group* Given a walking goal of 10,000 steps a day and information related to the benefits of exercise; instructed to use the app to achieve and monitor the goal *Control group* Given a walking goal of 30 min a day and information related to the benefits of exercise *Multi-component versus stand-alone app intervention* Stand-alone	*Outcome* Physical activity (steps/day) Other relevant outcomes None *Measures* Physical activity (Accupedo-Pro pedometer app)	*Physical activity* Significant between-group improvements in favour of the app intervention group (F1,53 = 4.30, *p* = 0.043, ηp2 = 0.08); significantly higher increase in steps in app intervention group (2393) compared to control group (1101; t53 = 2.07, *p* = 0.043. Significant within-group improvements for both intervention group (t27 = −6.14, *p* < .001) and control group (t26 = −2.25, *p* = .033). *Study quality* Fair CONSORT score: 12 Percentage of fulfilled criteria: 50.0%
Wharton et al. 2014 [42] USA	*Study design* 3-group RCT *Duration* Intervention exposure: 8 weeks Measurement points: baseline, 8 weeks Attrition rate: 18% *Sample* Adults *N* = 57 (47 analysed) 42.0 years/18–65 years 26% (M), 74% (F) Random (but controlled for sex, age and BMI)	*Behaviour change theory* Not reported *App features* Commercially available app: The ‘Lose It!’ app includes self-monitoring of dietary intake; performance feedback via daily calorie gauge graphic, calculated energy allotment and individual anthropometric data *Intervention group 1* Goal setting for weight loss, app group self-monitored dietary intake via an app diary (Lose It!), instruction to expend 150 calories/day via structured exercise, received a chart of physical activity options with approx. energy expenditures for 30, 40, 50 and 60 min. *Intervention group 2* Goal setting for weight loss, personally written diet plan, memo group self-monitored dietary intake via the memo function of their smartphone, face-to-face nutrition counselling sessions prior to the start of study, weekly emails to encourage healthy eating, instruction to expend 150 calories/day via structured exercise, received a chart of PA options with approx. energy expenditures for 30, 40, 50 and 60 min *Intervention group 3* Goal setting for weight loss, personally written diet plan, self-monitoring of dietary intake via paper and pencil notebook, personally written diet plan, face-to-face nutrition counselling sessions prior to the start of study, weekly emails to encourage healthy eating, instruction to expend 150 calories/day via structured exercise, received a chart of PA options with approx. energy expenditures for 30, 40, 50 and 60 min *Multi-component versus stand-alone app intervention* Stand-alone	*Outcome* Diet *Other relevant outcomes* Weight status (weight in pounds, BMI) *Measures* Diet (self-report, questionnaire: Healthy Eating Index) Weight status (questionnaire)	*Diet* No significant change in dietary intake. *Weight status* No significant difference in between-group change in weight, but significant within-group decrease in body weight in all groups (IG1: −3.5 ± 1.0, IG2: −6.5 ± 1.4, IG3: −4.4 ± 1.2; mean pounds). No significant change in BMI. *Study quality* Low CONSORT score: 10.5 Percentage of fulfilled criteria: 43.0%
Wang et al. 2015 [43] USA	*Study design* 2-group RCT *Duration* Intervention exposure: 6 weeks Measurement points: baseline, weeks 1, 2, 3, 4, 5, and 6 (Fitbit), 6 weeks (accelerometer) Attrition rate: 9% *Sample* Adults *N* = 67 49.3 years/18–69 years 9% (M), 91% (F) Random	*Behaviour change theory* Not reported *App features* Commercially available app: The Fitbit One Tracker that include self-monitoring through a wearable tracker and website/mobile app. *Intervention group* Daily SMS-based physical activity prompts plus self-monitoring with the FitBit One *Control group* Self-monitoring with the FitBit One *Multi-component versus stand-alone* *app intervention* Multi-component	*Outcomes* Physical activity (moderate-to-vigorous, total; steps/day and minutes/week) *Measures* Physical activity (accelerometer, Fitbit)	*Physical activity* Significant within-group increase in physical activity in IG at 1-week follow-up (steps/day: +1,266, SE: 491, *p* = 0.01; moderate-to-vigorous physical activity minutes/week: +17.8, SE: 8.5, *p* = 0.04; total physical activity: +38.3, SE: 15.9, *p* = 0.02). Significant within-group increase in moderate-to-vigorous physical activity minutes/week in CG (4.3; SE: 2.0; *p* = 0.04) at 6-week follow-up. However, the significant within-group changes were not maintained at the weeks 2–6 follow-ups. Moreover, no significant between-group changes in steps, as well as moderate-to-vigorous and total physical activity at 6-week follow-up. *Study quality* High CONSORT score: 17.5 Percentage of fulfilled criteria: 71.4%

*Abbreviations*: *M* male, *F* female, *IG* intervention group, *CG* control group

### Study quality assessment

The quality of the included studies was assessed using 25-point criteria adapted from the CONSORT checklists for the reporting of randomised controlled trials [26]. While the CONSORT checklist is intended for controlled trials, most criteria are applicable to other study designs and the weaker study designs justifiably received a lower score than studies using a controlled trial design. This approach has been used in other reviews [24]. Each criterion was rated as 1 (fulfilled), 0.5 (not all sub-items making up the criterion were fulfilled), 0 (not fulfilled or unclear), or not applicable (criterion was not applicable to the study design). Not applicable criteria were discounted from the ‘overall study quality score’ (sum of points). Hence, the highest attainable quality score was not 25 for all studies. Adapted from previous reviews [27, 28], the obtained study quality score for each study was divided by the highest attainable score and multiplied by 100 to give a percentage of fulfilled criteria; and studies were then grouped into high (>66.7%), fair (50–66.6%) or low (<50%) study quality (Additional file 3). The study quality assessment was conducted independently by two reviewers (SS, SA), with disagreement resolved by discussion and consensus with a third reviewer (WVL). Percent agreement between reviewers one and two for the scoring of the CONSORT criteria was 89%, with the most common points of discrepancy relating to recruitment methods, outcomes reporting and blinding procedures.

## Results

### Study selection

**Fig. 1:**
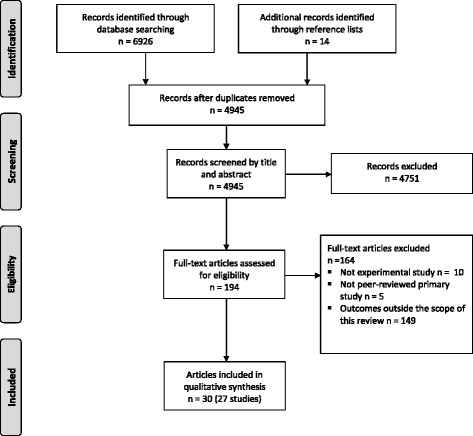
Flowchart of study selection process

A flowchart of the study selection process is presented in Fig. 1. A total of 6926 publications were identified from the database search. After removal of duplicates, 4945 publication titles and abstracts were screened, and 194 full-text articles were considered potentially eligible for inclusion. Of these, 30 articles reporting data on the efficacy of an app-based intervention to improve diet, physical activity and/or sedentary behaviour for prevention were included for final review. Some articles [29–34] reported on the same studies, and as such, a total of 30 articles describing 27 studies were included in this review.

### Study characteristics

Characteristics of the app intervention studies included in this review are presented in Table 1.

Twelve studies were conducted in North America [35–46], seven studies in Australia/New Zealand [29–32, 47–51], seven studies in Europe [33, 34, 52–57], and one study in the Middle-East [58]. Most studies were randomised controlled trials (*n* = 19) with 2-group [29, 31, 35, 40, 43–46, 48, 50, 52–55, 58] or 3-group [33, 34, 42, 47, 56, 57] study designs. The remaining studies were controlled trials (*n* = 3) [33, 37, 49], randomised trials (*n* = 1) [39] or pre-post studies (*n* = 4) [36, 38, 41]. Twenty-three studies [31–36, 38, 39, 41–46, 48–58] targeted adults (mean age: 41.5 years, range: 18–71 years) and four studies [29, 37, 40, 47] targeted children or adolescents (mean age: 12.4 years, range: 8–17 years). The total number of participants across the 27 studies was 2699 (510 children/adolescents, 2189 adults). Samples sizes ranged from 17 to 502 (mean sample size: 100). The duration of interventions ranged from 1–24 weeks, with an average intervention duration of 10 weeks. Follow-up assessments were at 4 weeks (*n* = 5), 8 weeks (*n* = 8), 12 weeks (*n* = 9), 20 weeks (*n* = 2), 6 months (*n* = 2), 9 months (*n* = 1) and 18 months (*n* = 1). Attrition rates ranged from 0% [36] to 57% [51], with an average attrition rate of 17%. The majority of studies [31, 33, 35–45, 47, 48, 50, 53, 55–58] reported higher rates of female participation (on average 64% of participants were female). The targeted health behaviours were dietary intake (*n* = 13), physical activity (*n* = 21) and sedentary behaviour (*n* = 5). Other reported lifestyle-related health outcomes were: weight status (*n* = 11); fitness (*n* = 3); blood pressure (*n* = 4); blood glucose (*n* = 1); cholesterol (*n* = 1); and quality of life (*n* = 2). Ten studies [33, 36–38, 43–45, 49, 50, 55, 57] targeted a single health behaviour, whilst 17 studies [29, 31, 35, 39–42, 46–48, 50–56, 58] targeted multiple health behaviours and related health outcomes. Fourteen studies [33, 37–40, 42, 45–47, 51, 52, 55–57] involved interventions delivered solely via an app (stand-alone intervention) and 13 studies [29, 31, 35, 36, 41, 43, 44, 48–50, 53, 54, 58] involved interventions that used apps in conjunction with other intervention strategies (multi-component intervention), such as physical education, parental education, counselling sessions, printed materials, motivational emails, websites and pedometer use. Fifteen studies [29, 31, 36–39, 45, 46, 48–50, 52, 56–58] used a newly designed app in the intervention and 12 studies [33, 35, 40–44, 47, 51, 53–55] used a commercially available app. Further, 15 of the 27 studies reported intervention designs based on behaviour change theories, such as Self-determination Theory (*n* = 3) [29, 35, 37], Transtheoretical Model (*n* = 4) [31, 33, 41, 48], Social Cognitive Theory (*n* = 4) [29, 39, 44, 46], Theory of Planned Behaviour (*n* = 2) [45, 50], Control Systems Theory of Self-regulation (*n* = 1) [58], and the Behaviour Change Wheel (*n* = 1) [55].

### Study quality

A detailed summary of quality assessments of included studies is presented in the Additional file 3. Overall, study quality ranged from high (*n* = 11) [29, 31, 43–45, 47, 48, 50, 53, 54, 58], to fair (*n* = 8) [35, 37, 46, 49, 55–57], and low (*n* = 8) [33, 36, 38, 39, 41, 42, 51, 52]. Study quality of interventions targeted to children/adolescents was high (*n* = 2) [29, 47] and fair (*n* = 2) [37, 40], and study quality of interventions targeted to adults ranged from high (*n* = 9) [31, 44, 45, 48, 50, 53, 54, 58], to fair (*n* = 6) [35, 46, 49, 55–57], and low (*n* = 8) [33, 36, 38, 39, 41, 42, 51, 52]. Most of the 13 interventions that used an app in combination with other intervention strategies were of high quality (*n* = 9) [29, 31, 43, 44, 48, 50, 53, 54, 58], whilst most of the 14 stand-alone app interventions were of fair (*n* = 6) [37, 40, 46, 55–57] or low quality (*n* = 6) [33, 38, 39, 42, 51, 52]. Study quality did not differ markedly between app interventions targeting multiple health behaviours and related health outcomes (high: *n* = 8, fair: *n* = 4, low: *n* = 5) and those targeting a single health behaviour (high: *n* = 3, fair: *n* = 4, low: *n* = 3). On average, the included studies fulfilled 61% of the assessment criteria (range: 34–92%). Most studies met the CONSORT requirements to provide a strong scientific rationale and described their participant eligibility, statistical methods and interventions clearly. Fewer studies reported sample size calculations [29, 31, 35, 43, 47, 50, 53–55, 57, 58] and included randomisation [29, 31, 35, 37, 39, 43–48, 50, 52–55, 58] and blinding procedures [31, 44–50, 53] in their study design. Attrition rates were reported or could be calculated for the majority of studies [29, 31, 33, 35–51, 53–58].

### Intervention efficacy

A summary of intervention effects for the included lifestyle behaviour outcomes (diet, physical activity, sedentary behaviour) and related health outcomes (weight status, fitness, blood pressure, glucose, cholesterol, quality of life) are presented in Table 2. Overall, a slightly larger proportion of single health behaviour interventions (5 out of 10; 50%) [33, 45, 49, 55, 57] showed significant between-group improvements than multiple health behaviour interventions (7 out of 17; 41%) [29, 31, 48, 50, 53, 54, 58]. Further, a larger proportion of interventions that used an app in conjunction with other intervention strategies (8 out of 13; 62%) demonstrated significant between-group improvements in the behavioural and health outcomes [29, 31, 48–50, 53, 54, 58] compared to stand-alone app interventions (5 out of 14; 36%) [33, 40, 45, 55, 57].

**Table 2:** Summary of intervention effects on behaviour outcomes and related health outcomes

	Behaviour outcomes			Related health outcomes					
Study	Diet	Physical activity	Sedentary behaviour	Weight status	Fitness	Blood pressure	Glucose	Cholesterol	Quality of life
Children									
Direito et al. 2015 [47]		0	0		0				
Garde et al. 2015 [37]		+ (w)							
Nollen et al. 2014 [40]	0		0	0					
Smith et al. 2014 [29], Lubans et al. 2016 [30]	+ (b)	0	+ (b)	0	+ (b)				
Adults									
Choi et al. 2016 [44]		0							
Cowdery et al. 2015 [35]		0		0		0			
Elbert et al. 2016 [57]	+ (b)								
Fukuoka et al. 2010 [36]		+ (w)							
Gasser et al. 2006 [52]	0	0		0					
Gilliland et al. 2015 [38]	+ (w)								
Gilson et al. 2016 [51]	0	0							
Glynn et al. 2014 [53]		+ (b)		0		0			0
Hebden et al. 2014 [48]	0	+ (b)	0	0					
King et al. 2013 [39]		+ (w)	+ (w)						
Kirwan et al. 2012 [49]		+ (b)							
Maher et al. 2015 [50]		+ (b)							0
Mummah et al. 2016 [45]	+ (b)								
Partridge et al. 2015 [31], Allman-Farinelli et al. 2016 [32]	+ (b)	+ (b)		+ (b)					
Rabbi et al. 2015 [46]	0	0							
Rospo et al. 2016 [56]		- (b)			+ (w)	+ (w)			
Safran Naimark et al. 2015 [58]	+ (b)	+ (b)		+ (b)					
Silveira et al. 2013 [33], Van Het Reve et al. 2014 [34]		+ (b)							
Stuckey et al. 2011 [41]		+ (w)		+ (w)		+ (w)	0	+ (w)	
Van Drongelen et al. 2014 [54]	+ (b)	+ (b)							
Wharton et al. 2014 [42]	0			+ (w)					
Walsh et al. 2016 [55]		+ (b)							
Wang et al. 2015 [43]		+ (w)							

+ (b): between-group significant improvements in favour of app intervention group, − (b): between-group significant improvements in favour of non-app control group, + (w): within-group significant improvement, 0: no significant change

### Children and adolescents

Of the four studies that specifically targeted children and/or adolescents, one study [29] reported significant between-group improvements in diet, sedentary behaviour and fitness in the app intervention group. Another study [37] reported a significant within-group increase in physical activity, but no significant difference between groups. The remaining two studies [40, 47] reported no significant changes in the behavioural or related health outcomes.

### Adults

Of the 23 studies that targeted adults, 17 studies reported significant improvements in diet (*n* = 6) [31, 38, 45, 54, 57, 58], physical activity (*n* = 13) [31, 33, 36, 37, 39, 41, 43, 48–50, 53–55, 58], sedentary behaviour (*n* = 1) [39], and other improved outcomes including weight status (*n* = 4) [31, 41, 42, 58], 49], fitness (*n* = 1) [56], blood pressure (*n* = 2) [41, 56] and cholesterol (*n* = 1) [41]. Of the studies reporting significant findings, 11 studies detected significant between-group differences in diet (*n* = 5) [31, 45, 54, 57, 58], physical activity (*n* = 9) [31, 33, 48–50, 53–55, 58] and weight status (*n* = 2) [31, 58] in favour of the app intervention group. Seven studies found significant within-group improvements in diet (*n* = 1) [38], physical activity (*n* = 4) [33, 36, 37, 39, 41, 43], sedentary behaviour (*n* = 1) [39] and weight status (*n* = 2) [41, 42, 56], blood pressure (*n* = 2) [41, 56] and cholesterol levels (*n* = 1) [41]. Five studies [35, 44, 46, 51, 52] reported no significant changes in the health outcomes of interest, and no significant findings were found in relation to the outcome glucose levels (assessed in one study).

### Characteristics of efficacious interventions

App interventions showing significant between-group improvements in the behavioural and health outcomes tended to be multi-component interventions [31, 36, 41, 43, 48–50, 53, 54, 58], with sample sizes above 90 participants [35, 43, 44, 47–49, 53] and intervention durations longer than 8 weeks [37, 42–45, 47, 49]. A slightly larger proportion of single health behaviour [33, 45, 49, 55, 57] versus multiple health behaviour interventions [29, 31, 48, 50, 53, 54, 58] demonstrated between-group improvements (50% versus 41%, respectively). Further, most of the interventions [29, 31, 33, 39, 42, 45, 48–50, 53, 55, 56, 58] showing significant improvements in the behavioural and health outcomes included goal-setting, self-monitoring and performance feedback in the app design. Some efficacious interventions also incorporated other behaviour change techniques, such as motivational messages [29, 36, 57], health education/tailored advice [29, 31, 38, 48, 54, 57], reinforcement [33, 39, 40, 45], gamification in the form of exergames, award and rewards [37–40, 50], social support through interaction with peers [33, 37, 50] and friendly team challenges [29, 37, 39, 45]. There was not enough data to identify which behaviour change techniques determined intervention efficacy. Moreover, there was no difference in the behaviour change techniques incorporated in apps for children compared to those used in apps for adults. Eleven studies [31, 38, 40, 43, 45, 48–50, 54, 56–58] out of the 19 studies showing significant improvements in behavioural and health outcomes reported usage statistics to determine participants’ engagement with the app. Three of these studies [38, 43, 49] examined associations between app usage and changes in the behavioural and health outcomes. Their findings showed that higher app usage was associated with improvements in physical activity and healthy eating [38, 43, 49].

## Discussion

This systematic review found modest evidence for the efficacy of app interventions to improve diet, physical activity and sedentary behaviours for NCD prevention. Overall, 19 out of the 27 identified studies reported significant improvements in behavioural and related health outcomes. Most of these studies reported significant between-group improvements in the app intervention group versus comparison group, which is considered the gold standard to demonstrate intervention efficacy [59]. Notwithstanding study limitations, the findings from this review indicate that apps can be an effective tool to improve health behaviours. The advantages of smartphone apps over other intervention delivery modes such as websites, face-to-face counselling and group sessions may partially explain the efficacy of app interventions. Given that many people have busy lifestyles, they value convenient access to health behaviour change programs that provide information and advice, real-time self-monitoring, feedback, reinforcement, social support, and rewards ‘on the go’ [60]. The appeal of smartphones for assistance in health promotion concurs with the trend that more people are seeking health information via mobile devices [61, 62]. In this context, apps provide the opportunity to bring behavioural interventions into real life situations where people make decisions about their health.

Despite the potential of apps, half of the interventions identified in this review used apps in conjunction with other intervention strategies. The remaining interventions were stand-alone interventions where the app was the sole intervention component. Importantly, however, is the observation that most of the multi-component interventions demonstrated significant between-group improvements in behavioural and health outcomes, whereas fewer stand-alone app interventions reported significant between-group improvements. This raises the question whether multi-component interventions yield stronger intervention effects than stand-alone app interventions. Many reviews of health behaviour change interventions [63–67] recommend the use of multiple intervention strategies to achieve long-term health behaviour change. Reviews of website-delivered interventions [9, 28, 68] have also shown stronger behaviour change effects when combined with other intervention strategies such as SMS, telephone coaching and motivational emails. As such, it is likely that the integration of apps in multi-component interventions produces stronger health outcomes compared to stand-alone app interventions; however, this remains to be tested in future trials.

The majority of app interventions were targeted to adults; only four out of the 27 identified app interventions were specifically aimed at children or adolescents. This is consistent with previous e & mHealth reviews [11, 12, 25, 69] showing that internet and mobile phone delivered interventions have mainly targeted adult populations. Although smartphones and tablets have become an integral part of children’s lives [70], surprisingly few interventions have yet utilised app technology in pediatric health behaviour change programs [12]. However, as exergame and serious game apps are becoming increasingly popular among children and adolescents [71], it is likely that more app studies focussing on children will be conducted in the near future.

The average attrition rate (i.e., participant loss to follow-up) reported in the app interventions was 17%, which is lower compared average attrition rates of 23–27% found in web-based interventions [28, 68, 72]. Lower participant attrition indicates less bias in the estimated intervention effects [73]. However, participant attrition does not capture participants’ engagement with the app intervention which also determines intervention effects. For example, web- and app-based interventions that have examined participant engagement found that higher levels of website and app usage were associated with increased intervention efficacy [38, 43, 68]. Despite this, less than half of the studies reported usage statistics to determine participants’ engagement with the app. This is consistent with previous reviews [24, 28, 68] reporting that few internet and mobile interventions recorded participants’ engagement with the intervention technologies. Given the relative ease by which app usage statistics can be tracked, it is unfortunate that this data is not being collected and published for all studies in this area. We know that participant engagement, measured by number of website logins, usually declines after the first few weeks in website and social media interventions [24, 28, 68]. An example is the Australian 10,000 Steps program, a successful, freely available web- and app-based intervention to promote physical activity [61]. Participants’ engagement with the 10,000 Steps website and app lasts on average 5–6 weeks, with longer usage duration (on average 8 weeks) being observed in people who use both the 10,000 Steps website and app [61]. This rapid decline in usage concurs with qualitative research showing that people often lack commitment to using any particular app and they tend to engage in only transient, casual app use [60]. Since participant engagement determines intervention exposure, and level of intervention exposure determines intervention efficacy [28, 61, 74], better understanding of factors that improve participant engagement and retention is needed.

To increase user engagement with health behaviour change apps, more information is needed about what app features and behaviour change techniques people value and use. Recent focus group data showed that [60, 75] that young people value health behaviour apps that require low effort, are pleasant to use, are developed by credential experts, enable self-monitoring, provide advice on how to change behaviour, include positively framed alerts/reminders (but not too frequent), provide accurate tracking functions, incorporate adequate privacy settings, and clearly show what the app will do (no surprises). Some of these user preferences were part of the efficacious apps identified in this review. For example, most apps included goal-setting, self-monitoring and performance feedback [29, 31, 33, 39, 42, 45, 48–50, 53, 55, 56, 58]. Additionally, some apps incorporated tailored advice, motivational prompts and reinforcement, gamification, social support or friendly team challenges [29, 33, 37–40, 45, 48, 50, 54, 57]. Reviews of web-based interventions have demonstrated that interventions including more behaviour change techniques are more effective [74, 76]. This may also apply to app-based interventions. However, it remains unclear what the optimal number and combination of app features and behaviour change techniques is to increase user retention and ultimately intervention efficacy. It is also possible that efficacy declines when too many features or techniques are implemented. Furthermore, socio-demographic factors (e.g., sex, age, education) and psychosocial factors (e.g., attitudes, perceived benefits, enjoyment) may also influence app usage [60]. Emerging research on the adoption of app technology showed that higher app usage is associated with being female and of younger age [61, 77], as well as with personal interest in new technologies, positive attitudes towards smartphone apps and perceived benefit of use [77]. Therefore, targeting and tailoring smartphones apps to specific population groups may also enhance the efficacy of app-based interventions.

### Strengths and limitations

Strengths of this systematic review are that it was conducted and reported according to PRISMA guidelines [23], and study quality was systematically assessed using the CONSORT checklist [26]. The search strategy was comprehensive, and study selection, data extraction, and quality rating were completed by two independent reviewers, as is standard practice for high quality systematic review [23]. These procedures ensure accuracy of the reviewed data. The scope of this review was limited to app interventions improving behavioural and related health outcomes for prevention; hence app interventions relating to chronic disease treatment were not captured in this review. Few app interventions identified in this review focused on dietary and sedentary behaviours which makes it more difficult to draw conclusions on the efficacy of app interventions targeting these behaviours, as opposed to those targeting physical activity behaviour. Furthermore, the included studies varied widely in terms of methodological quality, with some studies scoring very poorly, thereby reducing the trust that can be placed in their findings. Finally, the possibility of publication bias should also be acknowledged. As with all systematic reviews examining the efficacy of interventions, it is possible that some studies with null findings have not been published [78].

### Recommendations for future research

Based on this review, it is recommended that future studies:

- Test the efficacy of specific app features and behaviour change techniques in high quality controlled trials to distinguish effective from ineffective intervention components.
- Directly compare the efficacy of stand-alone app intervention compared to multi-component interventions that use apps in combination with other intervention strategies.
- Compare the efficacy of app interventions to other intervention delivery modes, such as website, print-based and face-to-face interventions.
- Utilise larger sample sizes to ensure they are sufficiently powered to detect significant intervention effects.
- Tailor app interventions to specific population groups (e.g., women, young people) in whom usage and adoption of app technology is high.
- Report app usage statistics using objective and self-report measures to examine levels of and reasons for participant (dis)engagement and intervention exposure.
- Explore the optimal duration and intensity of app interventions to ensure user engagement and retention as the intervention progresses.
- Identify factors that increase user engagement and retention in app interventions to sustain behavioural health improvments in the long-term.
- Investigate the relationship between user engagement and intervention efficacy, whilst taking into account socio-demographic and psychosocial factors.

## Conclusions

Interventions using apps to improve diet, physical activity and sedentary behaviour for prevention show promise for effective behaviour change in children and adults. The evidence base is largest for the use of apps to increase physical activity in adults. Fewer interventions have used apps to improve dietary and sedentary behaviours, and very few app interventions have targeted children and adolescents. Multi-component interventions that combine apps with other intervention strategies appear to be more effective than stand-alone app interventions, however, this remains to be tested further in controlled trials. Overall, there is still considerable scope to improve the efficacy of app-based interventions. In particular, intervention studies should gather more app usage statistics to identify factors that improve user engagement and retention, and its relationship with intervention efficacy. In addition, more formative research is needed to determine the optimal number and combination of app features, behaviour change techniques, and level of participant contact needed to maximise user engagement and ultimately intervention efficacy.

## Additional files

Additional file 1: Completed PRISMA checklist. (DOCX 77 kb)
Additional file 2: Search strategy used in the databases. (DOCX 32 kb)
Additional file 3: Quality assessment for all included studies. (DOCX 20 kb)

## Abbreviations

Apps: Applications
CONSORT: Consolidated standards of reporting trials
NCDs: Non-communicable diseases
PRISMA: Preferred reporting items for systematic reviews and meta-analyses
