# Supplementary material for: Efficacy of interventions that use apps to improve diet, physical activity and sedentary behaviour: a systematic review
Source: Int J Behav Nutr Phys Act. 2016 Dec 7;13:127. doi: 10.1186/s12966-016-0454-y (PMC5142356; doi:10.1186/s12966-016-0454-y)
Supplement: Additional file 1: — Completed PRISMA checklist. (DOCX 77 kb) [file 12966_2016_454_MOESM3_ESM.docx]

Additional file 3: Quality assessment for all included studies

|  | Smith et al 2014 | Direito et al 2015 | Glynn et al 2014 | Safran Naimark et al 2015 | Fukuoka et al 2010 | King et al 2013 | Gasser et al 2015 | Cowdery et al 2015 | Hebden et al 2015 | Kirwan et al 2015 | Silveira et al 2013, Van het Reve et al 2014 | Van Drongelen et al 2012 | Wharton et al 2014 | Nollen et al 2014 | Gilliland et al 2015 | Wang et al 2015 | Stuckey et al 2011 | Garde et al 2015 | Maher et al 2015 |
| --- | --- | --- | --- | --- | --- | --- | --- | --- | --- | --- | --- | --- | --- | --- | --- | --- | --- | --- | --- |
| **Title and abstract** |  |  |  |  |  |  |  |  |  |  |  |  |  |  |  |  |  |  |  |
| a) identification as randomized trial in title; b) structured summary | 1 | 1 | 1 | 1 | 0.5 | 0.5 | 0.5 | 0.5 | 1 | 0.5 | 0.5 | 0.5 | 0.5 | 1 | 0.5 | 1 | 0.5 | 0.5 | 1 |
| **Introduction** |  |  |  |  |  |  |  |  |  |  |  |  |  |  |  |  |  |  |  |
| a) scientific background/rationale; b) specific objectives/ hypotheses | 1 | 1 | 1 | 1 | 1 | 1 | 1 | 1 | 1 | 1 | 1 | 1 | 1 | 1 | 1 | 1 | 1 | 1 | 1 |
| **Methods** |  |  |  |  |  |  |  |  |  |  |  |  |  |  |  |  |  |  |  |
| **T*rial design***  a) description of trial design; b) changes in methods after trial commencement | 0.5 | 0.5 | 0.5 | 0.5 | 0.5 | 0.5 | 0 | 0.5 | 0.5 | 0.5 | 0.5 | 1 | 0.5 | 0.5 | 0.5 | 0.5 | 0.5 | 0.5 | 0.5 |
| ***Participants*** |  |  |  |  |  |  |  |  |  |  |  |  |  |  |  |  |  |  |  |
| a) eligibility criteria; b) settings and locations of data collection | 1 | 1 | 1 | 1 | 1 | 0.5 | 0.5 | 1 | 1 | 1 | 1 | 1 | 0.5 | 1 | 0.5 | 1 | 1 | 1 | 1 |
| ***Interventions*** |  |  |  |  |  |  |  |  |  |  |  |  |  |  |  |  |  |  |  |
| Descriptions of sufficient details to allow replication | 1 | 1 | 0 | 1 | 1 | 1 | 1 | 1 | 1 | 1 | 1 | 1 | 1 | 1 | 1 | 1 | 1 | 1 | 1 |
| ***Outcomes*** |  |  |  |  |  |  |  |  |  |  |  |  |  |  |  |  |  |  |  |
| a) pre-specified primary and secondary outcomes; b) changes to outcomes after trial commencement | 0.5, NA | 0.5 | 0.5 | 0.5 | 0.5 | 0.5 | 0.5 | 0.5 | 0.5 | 0.5 | 0.5 | 0.5 | 0 | 0.5 | 0.5 | 0.5 | 0.5 | 0.5 | 0.5 |
| ***Sample size*** |  |  |  |  |  |  |  |  |  |  |  |  |  |  |  |  |  |  |  |
| a) how sample size was determined; b) if applicable, interim analysis/ stopping guidelines | 1 | 0.5 | 0.5 | 0.5 | 0 | 0 | 0 | 0.5 | 0 | 0 | 0 | 0.5 | 0 | 0 | 0 | 0.5 | 0 | 0 | 0.5 |
| ***Randomization – sequence generation*** |  |  |  |  |  |  |  |  |  |  |  |  |  |  |  |  |  |  |  |
| a) method used; b) type of randomization including any type of restriction | 1 | 1 | 1 | 1 | 0 | 1 | 0.5 | 1 | 1 | 0 | 0 | 1 | 0 | 0 | 0 | 1 | 0 | 1 | 1 |
| ***Allocation concealment mechanism*** |  |  |  |  |  |  |  |  |  |  |  |  |  |  |  |  |  |  |  |
| Implementation of random allocation sequence, including concealment | 1 | 1 | 1 | 0 | 0 | 0 | 0 | 0 | 1 | 0 | 0 | 0 | 0 | 0 | 0 | 1 | 0 | 0 | 1 |
| ***Implementation*** |  |  |  |  |  |  |  |  |  |  |  |  |  |  |  |  |  |  |  |
| Who generated random allocation sequence, who enrolled participants, who assigned participants | 1 | 1 | 1 | 0 | 0 | 0 | 0 | 0 | 1 | 0 | 0 | 0 | 0 | 0 | 0 | 1 | 0 | 0 | 0 |
| ***Blinding*** |  |  |  |  |  |  |  |  |  |  |  |  |  |  |  |  |  |  |  |
| a) if done, who was blinded and how; b) if relevant, similarity of interventions | 0, NA | 1 | 0.5 | 0 | 0 | 0.5 | 0 | 0, NA | 0.5 | 0.5 | 0 | 0 | 0, NA | 0 | 0 | 0 | 0 | 1 | 0.5,NA |
| ***Statistical methods*** |  |  |  |  |  |  |  |  |  |  |  |  |  |  |  |  |  |  |  |
| Statistical methods used a) for primary outcomes; b) additional analyses | 1 | 1 | 0.5 | 1 | 1 | 1 | 0 | 1 | 1 | 1 | 1 | 1 | 0.5 | 0.5 | 1 | 1 | 0.5 | 1 | 1 |
| **Results** |  |  |  |  |  |  |  |  |  |  |  |  |  |  |  |  |  |  |  |
| ***Participant flow***  a) number of participants randomized, receiving treatment, and analyzed; b) losses and exclusions, with reasons | 1 | 1 | 1 | 1 | 0.5 | 0.5 | 0 | 0 | 1 | 0.5 | 1 | 1 | 1 | 1 | 0.5 | 1 | 1 | 1 | 1 |
| ***Recruitment*** |  |  |  |  |  |  |  |  |  |  |  |  |  |  |  |  |  |  |  |
| a) dates of recruitment and follow-up; b) why the trial ended | 0.5, NA | 0.5, NA | 0.5, NA | 0.5, NA | 0.5,NA | 0.5,NA | 0 | 0 | 0.5, NA | 0.5, NA | 0 | 0 | 0 | 0.5 | 0, NA | 0.5 | 0.5 | 0.5, NA | 0.5 |
| ***Baseline data*** |  |  |  |  |  |  |  |  |  |  |  |  |  |  |  |  |  |  |  |
| A table with baseline demographic and clinical characteristics for each group | 1 | 1 | 1 | 1 | 0 | 0 | 0 | 1 | 1 | 1 | 1 | 1 | 1 | 1 | 0 | 1 | 0 | 1 | 1 |
| ***Numbers analyzed*** |  |  |  |  |  |  |  |  |  |  |  |  |  |  |  |  |  |  |  |
| For each group, number of participants included in each analyses | 1 | 1 | 1 | 1 | 1 | 1 | 1 | 1 | 1 | 1 | 1 | 1 | 0 | 0 | 1 | 1 | 1 | 1 | 1 |
| ***Outcomes and estimation*** |  |  |  |  |  |  |  |  |  |  |  |  |  |  |  |  |  |  |  |
| a) results for each group, and the estimated effect size and its precision; b) absolute and relative effect sizes for binary outcomes | 1 | 0.5, NA | 0 | 0.5 | 0 | 0 | 0.5 | 0.5,NA | 0.5 | 0.5 | 0.5 | 0.5 | 0.5,NA | 0.5,NA | 0 | 0.5, NA | 0, NA | 0 | 0.5 |
| ***Ancillary analyses*** |  |  |  |  |  |  |  |  |  |  |  |  |  |  |  |  |  |  |  |
| Results of any other analyses performed, distinguishing pre-specified from exploratory | 1 | 1 | 0 | 1 | 0 | 1 | 1 | 1 | 1 | 1 | 0 | 1 | 1 | 1 | 1 | 0 | 0 | 1 | 1 |
| ***Harms*** |  |  |  |  |  |  |  |  |  |  |  |  |  |  |  |  |  |  |  |
| Harms or unintended effects in each group | 1 | 1 | 0 | 0 | 0 | 0 | 0 | 0 | 0 | 0 | 0 | 0 | 0 | 0 | 0 | 0 | 0 | 0 | 0 |
| **Discussion** |  |  |  |  |  |  |  |  |  |  |  |  |  |  |  |  |  |  |  |
| ***Limitations***  Trial limitations/ bias/ multiplicity of analyses | 1 | 1 | 1 | 1 | 1 | 1 | 1 | 1 | 1 | 1 | 1 | 1 | 1 | 1 | 0 | 1 | 1 | 1 | 1 |
| ***Generalisability*** |  |  |  |  |  |  |  |  |  |  |  |  |  |  |  |  |  |  |  |
| Generalisability (external validity/ applicability) of findings | 1 | 0 | 1 | 1 | 1 | 0 | 0 | 1 | 1 | 1 | 0 | 1 | 0 | 0 | 0 | 1 | 0 | 1 | 1 |
| ***Interpretation*** |  |  |  |  |  |  |  |  |  |  |  |  |  |  |  |  |  |  |  |
| Consistent with results and balanced | 1 | 1 | 1 | 1 | 1 | 1 | 0 | 1 | 1 | 1 | 1 | 1 | 1 | 1 | 1 | 1 | 1 | 1 | 1 |
| **Other information** |  |  |  |  |  |  |  |  |  |  |  |  |  |  |  |  |  |  |  |
| ***Registration***  Registration number and name of registry | 0 | 1 | 1 | 1 | 0 | 0 | 0 | 0 | 0 | 0 | 0 | 1 | 0 | 0 | 0 | 0 | 0 | 0 | 1 |
| ***Protocol*** |  |  |  |  |  |  |  |  |  |  |  |  |  |  |  |  |  |  |  |
| Where full trial protocol can be accessed | 1 | 1 | 1 | 0 | 0 | 0 | 0 | 0 | 1 | 0 | 0 | 1 | 0 | 0 | 0 | 0 | 0 | 0 | 0 |
| ***Funding*** |  |  |  |  |  |  |  |  |  |  |  |  |  |  |  |  |  |  |  |
| Sources of funding/ role of funders | 1 | 1 | 1 | 0 | 1 | 1 | 1 | 0 | 0 | 1 | 1 | 0 | 1 | 1 | 0 | 1 | 1 | 1 | 1 |
| **Study quality score attainable** | 23.5 | 24 | 24.5 | 24.5 | 24.5 | 24.5 | 25 | 24 | 24.5 | 24.5 | 25 | 25 | 24 | 24.5 | 24.5 | 24.5 | 24.5 | 24.5 | 24.5 |
| **Study quality score** | 21.5 | 21 | 18 | 16.5 | 11.5 | 12 | 8.5 | 13.5 | 19.5 | 14.5 | 12 | 17 | 10.5 | 12.5 | 8.5 | 17.5 | 11 | 16 | 19 |
| **Study quality percentage** | 91.5 | 87.5 | 73.5 | 67.3 | 46.9 | 46.9 | 34.0 | 56.3 | 79.6 | 59.2 | 48.0 | 68.0 | 43.0 | 51.0 | 34.7 | 71.4 | 45.0 | 65.3 | 77.6 |
| **Study quality rating** | high | high | high | high | low | low | low | fair | high | fair | low | high | low | fair | low | high | low | fair | high |

Additional file 2: Quality assessment for all included studies - continued

|  | Partridge et al. 2015;  Allman-Farinelli et al.  2016 | Choi et al. 2016 | Elbert et al. 2016 | Gilson et al. 2016 | Rabbi et al. 2015 | Mummah et al. 2016 | Rospo et al. 2016 | Walsh et al. 2016 |
| --- | --- | --- | --- | --- | --- | --- | --- | --- |
| **Title and abstract** |  |  |  |  |  |  |  |  |
| a) identification as randomized trial in title; b) structured summary | 1 | 1 | 1 | 0.5 | 1 | 1 | 0.5 | 0.5 |
| **Introduction** |  |  |  |  |  |  |  |  |
| a) scientific background/rationale; b) specific objectives/ hypotheses | 1 | 1 | 1 | 0.5 | 1 | 1 | 1 | 1 |
| **Methods** |  |  |  |  |  |  |  |  |
| **T*rial design***  a) description of trial design; b) changes in methods after trial commencement | 1 | 0.5 | 0.5 | 0.5 | 0.5 | 0.5 | 0.5 | 0.5 |
| ***Participants*** |  |  |  |  |  |  |  |  |
| a) eligibility criteria; b) settings and locations of data collection | 1 | 1 | 1 | 0.5 | 1 | 1 | 1 | 1 |
| ***Interventions*** |  |  |  |  |  |  |  |  |
| Descriptions of sufficient details to allow replication | 1 | 1 | 1 | 0 | 1 | 1 | 1 | 1 |
| ***Outcomes*** |  |  |  |  |  |  |  |  |
| a) pre-specified primary and secondary outcomes; b) changes to outcomes after trial commencement | 0.5 | 0.5 | 0.5 | 0.5 | 0.5 | 0.5 | 0.5 | 0.5 |
| ***Sample size*** |  |  |  |  |  |  |  |  |
| a) how sample size was determined; b) if applicable, interim analysis/ stopping guidelines | 0.5 | 0 | 0.5 | 0 | 0 | 0 | 0 | 0.5 |
| ***Randomization – sequence generation*** |  |  |  |  |  |  |  |  |
| a) method used; b) type of randomization including any type of restriction | 1 | 1 | 0 | 0 | 1 | 1 | 0 | 1 |
| ***Allocation concealment mechanism*** |  |  |  |  |  |  |  |  |
| Implementation of random allocation sequence, including concealment | 1 | 1 | 0 | 0 | 0 | 0 | 0 | 0 |
| ***Implementation*** |  |  |  |  |  |  |  |  |
| Who generated random allocation sequence, who enrolled participants, who assigned participants | 1 | 0 | 0 | 0 | 0 | 1 | 0 | 0 |
| ***Blinding*** |  |  |  |  |  |  |  |  |
| a) if done, who was blinded and how; b) if relevant, similarity of interventions | 0.5 | 0.5 | 0 | 0 | 0.5 | 0.5 | 0 | 0 |
| ***Statistical methods*** |  |  |  |  |  |  |  |  |
| Statistical methods used a) for primary outcomes; b) additional analyses | 1 | 1 | 1 | 0.5 | 1 | 1 | 1 | 0.5 |
| **Results** |  |  |  |  |  |  |  |  |
| ***Participant flow***  a) number of participants randomized, receiving treatment, and analyzed; b) losses and exclusions, with reasons | 1 | 1 | 1 | 1 | 1 | 1 | 1 | 1 |
| ***Recruitment*** |  |  |  |  |  |  |  |  |
| a) dates of recruitment and follow-up; b) why the trial ended | 1 | 0.5, NA | 0.5, NA | 0, NA | 0, NA | 0.5, NA | 0, NA | 0, NA |
| ***Baseline data*** |  |  |  |  |  |  |  |  |
| A table with baseline demographic and clinical characteristics for each group | 1 | 1 | 0 | 0 | 0 | 1 | 1 | 1 |
| ***Numbers analyzed*** |  |  |  |  |  |  |  |  |
| For each group, number of participants included in each analyses | 1 | 1 | 1 | 1 | 1 | 1 | 1 | 1 |
| ***Outcomes and estimation*** |  |  |  |  |  |  |  |  |
| a) results for each group, and the estimated effect size and its precision; b) absolute and relative effect sizes for binary outcomes | 0.5, NA | 0.5, NA | 0.5, NA | 0.5, NA | 0.5, NA | 0.5, NA | 0.5, NA | 0.5, NA |
| ***Ancillary analyses*** |  |  |  |  |  |  |  |  |
| Results of any other analyses performed, distinguishing pre-specified from exploratory | 1 | 1 | 1 | 1 | 1 | 1 | 1 | 0 |
| ***Harms*** |  |  |  |  |  |  |  |  |
| Harms or unintended effects in each group | 0 | 0 | 0 | 0 | 0 | 0 | 0 | 0 |
| **Discussion** |  |  |  |  |  |  |  |  |
| ***Limitations***  Trial limitations/ bias/ multiplicity of analyses | 1 | 1 | 1 | 1 | 1 | 1 | 1 | 1 |
| ***Generalisability*** |  |  |  |  |  |  |  |  |
| Generalisability (external validity/ applicability) of findings | 0 | 0 | 0 | 0 | 0 | 0 | 0 | 0 |
| ***Interpretation*** |  |  |  |  |  |  |  |  |
| Consistent with results and balanced | 1 | 1 | 1 | 1 | 0 | 1 | 1 | 1 |
| **Other information** |  |  |  |  |  |  |  |  |
| ***Registration***  Registration number and name of registry | 1 | 1 | 1 | 0 | 1 | 1 | 0 | 0 |
| ***Protocol*** |  |  |  |  |  |  |  |  |
| Where full trial protocol can be accessed | 1 | 0 | 0 | 0 | 0 | 0 | 0 | 0 |
| ***Funding*** |  |  |  |  |  |  |  |  |
| Sources of funding/ role of funders | 1 | 1 | 1 | 1 | 1 | 1 | 0 | 0 |
| **Study quality score attainable** | 24.5 | 24 | 24 | 24 | 24 | 24 | 24 | 24 |
| **Study quality score** | 21 | 17.5 | 14.5 | 9.5 | 14 | 17.5 | 12 | 12 |
| **Study quality percentage** | 85.7 | 72.9 | 60.4 | 39.6 | 58.3 | 72.9 | 50.0 | 50.0 |
| **Study quality rating** | high | high | fair | low | fair | high | fair | fair |
