# Supplementary material for: Efficacy of interventions that use apps to improve diet, physical activity and sedentary behaviour: a systematic review
Source: Int J Behav Nutr Phys Act. 2016 Dec 7;13:127. doi: 10.1186/s12966-016-0454-y (PMC5142356; doi:10.1186/s12966-016-0454-y)
Supplement: Additional file 3: — Quality assessment for all included studies. (DOCX 20 kb) [file 12966_2016_454_MOESM2_ESM.docx]

Additional file 2 Search strategies used in the databases

General search terms used in the searches

| Search category | Search terms |
| --- | --- |
| App | application OR app OR smartphone OR smart phone OR tablet OR mobile games OR games |
| Health behaviours | physical activity OR walk OR physical fitness OR leisure activity OR motor activity OR exercise OR sport OR  sedentary OR sedentary behaviour OR sedentary behavior OR sitting OR screen time OR inactive OR  diet OR dietary OR nutrition OR nutritional OR healthy eating OR food OR fruit OR vegetable OR snack OR soft drink OR carbonated beverages |
| Limits | Humans  01/01/2006 – 31/10/2016  RCT, comparative studies, journal article, systematic review, meta-analysis  English  Title/abstract |

**Web of Science**

TOPIC: (application OR app OR smartphone OR smart phone OR tablet OR game) *AND* TOPIC: (physical activity OR walk OR physical fitness OR leisure activity OR motor activity OR exercise OR sport OR sedentary OR sedentary behaviour OR sedentary behavior OR sitting OR screen time OR inactive OR diet OR dietary OR nutrition OR nutritional OR healthy eating OR food OR fruit OR vegetable OR snack OR soft drink OR carbonated beverages) *AND* TOPIC: (intervention OR program OR programme OR health promotion OR prevention OR trial) *AND* TOPIC: (child OR adolescent OR adult OR youth)

Refined by: DOCUMENT TYPES: ( ARTICLE OR REVIEW )

Timespan=2006-2016

2786 hits

**SportDiscuss & PsychInfo**

Limiters - Peer Reviewed; Document Type: Journal Article, Review-Any; Published Date: 20060101-20161231; Publication Year: 2006-2016; Publication Type: Peer Reviewed Journal; English; Population Group: Human; Exclude Dissertations; English Abstract Available; Language: English; Publication Type: Academic Journal, Review

( application OR app OR smartphone OR smart phone OR tablet OR mobile games OR games ) AND ( physical activity OR walk OR physical fitness OR leisure activity OR motor activity OR exercise OR sport OR sedentary OR sedentary behaviour OR sedentary behavior OR sitting OR screen time OR inactive OR diet OR dietary OR nutrition OR nutritional OR healthy eating OR food OR fruit OR vegetable OR snack OR soft drink OR carbonated beverages ) AND ( intervention OR program OR programme OR health promotion OR prevention OR trial )

2663 hits

**CINAHL**

S1: application OR app OR smartphone OR smart phone OR tablet OR mobile game OR game

S2: physical activity OR walk OR physical fitness OR leisure activity OR motor activity OR exercise OR sport OR sedentary OR sedentary behaviour OR sedentary behavior OR sitting OR screen time OR inactive OR diet OR dietary OR nutrition OR nutritional OR healthy eating OR food OR fruit OR vegetable OR snack OR soft drink OR carbonated beverage

S3: intervention OR program OR programme OR health promotion OR prevention OR trial

S4: child OR adolescent OR youth OR adult

S1 AND S2 AND S3 AND S4

Limiters - English Language; Published Date: 20060101-20161231; Human; Language: English
Narrow by Language: - English
Search modes - Find all my search terms

1179 hits

**Scopus**

(physical activity OR walk OR physical fitness OR leisure activity OR motor activity OR exercise OR sport OR sedentary OR sedentary behaviour OR sedentary behavior OR sitting OR screen time OR inactive OR diet OR dietary OR nutrition OR nutritional OR healthy eating OR food) AND (application OR app OR smartphone OR smart phone OR tablet OR mobile game OR game )

not limits

298 hits
